# Age Trajectories of O_2_ Saturation and Levels of Serum Bicarbonate or End-Tidal CO_2_ Across the Life Course of Women and Men: Insights from EHR and PSG Data

**DOI:** 10.3390/biom15060884

**Published:** 2025-06-17

**Authors:** Leping Li, Min Shi, David M. Umbach, Zheng Fan

**Affiliations:** 1 Biostatistics and Computational Biology Branch, National Institute of Environmental Health Sciences, National Institutes of Health, Durham, NC 27709, USA; shi2@niehs.nih.gov (M.S.); umbach@niehs.nih.gov (D.M.U.); 2 Division of Sleep Medicine and Department of Neurology, The University of North Carolina at Chapel Hill, Chapel Hill, NC 27599, USA; zhengfan@med.unc.edu

**Keywords:** CO_2_, serum bicarbonate, pulse oximetry, polysomnogram, electronic health records, sex difference, menopause

## Abstract

To elucidate the changes in gas exchange across the life course, we estimated the age trajectories of O_2_ saturation, CO_2_ (as either end-tidal or serum bicarbonate), resting heart rate, and resting respiratory rate from age 2 yr onward in female and male patients separately. We utilized two sources’ data: electronic health records (EHR) representing ambulatory visits of approximately 53,000 individuals and sleep clinic polysomnogram (PSG) records representing an additional ~21,000. We used linear regression to estimate age-group-specific mean response levels for women and men. We compared estimated female–male differences between pre- and post-pubertal children and between pre- and post-menopausal periods among adults. Women between 15 and 45 years had higher O_2_ saturation and lower serum bicarbonate levels or end-tidal CO_2_ levels than men of similar ages. For O_2_ saturation and for both measures of CO_2_, the female–male difference was larger on average among adults at pre-menopausal ages than those at post-menopausal ages. Women had higher O_2_ saturation throughout their lives than men; however, the difference disappeared in the elderly. Women between menarche and menopause had significantly lower end-tidal CO_2_ and serum bicarbonate than men of similar ages. After menopause, however, women appeared to have higher mean levels of both end-tidal CO_2_ and serum bicarbonate than men.

## 1. Introduction

Important biological, cultural, and behavioral differences between the sexes are widely recognized [1,2,3,4]. These include crucial differences in disease prevalence, susceptibility, and progression [5,6,7,8,9,10,11,12]. Differences in respiratory function between men and women also exist. Anatomically and physiologically, women have smaller lungs and lower maximal expiratory capacity compared with men [13,14,15,16,17]. Pathologically, women tend to experience more dyspnea and exacerbations of respiratory conditions compared with men, whereas men are more likely to develop certain lung diseases, such as idiopathic pulmonary fibrosis and chronic obstructive pulmonary disease (COPD) [18,19,20].

The sex differences in respiratory function may be largely attributed to differences in the anatomy of the chest wall and in the levels of sex hormones such as estrogen and progesterone [13,14,15,16,17,21,22,23,24]. Progesterone is known to stimulate respiration [21,22,23,24,25]. Progesterone acts on the central steroid receptor to increase the sensitivity of the respiratory center to modulate the respiratory control system [24]. Besides sex, aging is also associated with physiological changes in the respiratory system as the expiratory flow rates decrease with aging [26,27,28].

The sex differences in respiratory function may also be reflected in differences in the levels of blood gasses. Respiration involves gas exchange in which oxygen (O_2_) moves from the lungs to the bloodstream to provide O_2_ to organs, while carbon dioxide (CO_2_) passes from the blood to the lungs to allow for CO_2_ elimination [29,30,31]. These processes happen in the alveoli and the network of capillaries located in the walls of the alveoli in the lungs. Arterial blood O_2_ saturation level can be conveniently monitored using a pulse oximetry device. Pulse oximetry is based on the principle that oxygenated hemoglobin is distinguishable from non-oxygenated hemoglobin [32,33]. Like blood O_2_, blood CO_2_ also exists in multiple forms. The total CO_2_ content in blood includes the serum bicarbonate (HCO_3_^−^) as well as other forms of carbon dioxide, i.e., dissolved CO_2_ and carbonic acid. In clinical settings, the serum total CO_2_ content is measured in lieu of serum bicarbonate [29,34] which accounts for approximately 95% of the total CO_2_ content in serum. Some of the bicarbonate in the blood is carried back to the lungs and exhaled. Exhaled CO_2_ can be measured as end-tidal CO_2_ or transcutaneous CO_2_ [35]. At normal physiological levels, the amount of exhaled CO_2_ is delicately controlled by the kidneys and lung to maintain acid–base balance [36]. Available evidence has established that the concentration of exhaled CO_2_ correlates to the arterial concentration of CO_2_, although multiple factors can impact the relationship [37].

Arterial O_2_ saturation measured by oximetry indicates that O_2_ level decreases linearly with age [38]; however, whether the age trend differs between women and men is less known. Conversely, evidence for differences in the age trends of serum bicarbonate levels between the sexes is starting to emerge [39,40]. One study showed that the end-tidal CO_2_ levels also decreased with age [41]. We could, however, find no reports comparing the trajectories of both O_2_ and CO_2_ levels (either end-tidal or serum bicarbonate) between men and women across the lifespan.

In this manuscript, we aimed to fill that gap by examining the age trajectories in both O_2_ and CO_2_ levels between men and women using two independent datasets—pulse oximetry O_2_ saturation and serum bicarbonate level from ambulatory clinical visits and pulse oximetry O_2_ saturation and end-tidal CO_2_ levels from overnight in-laboratory polysomnography studies. In addition, we examined a possible role for progesterone’s influence on these trajectories by showing that the approximate ages of puberty and menopause are associated with changes in the between-sex differences in those gas levels.

## 2. Materials and Methods

### 2.1. Study Design

This study aimed to assess trajectories in O_2_, CO_2_ (in the form of end-tidal and serum bicarbonate), resting heart rate (HR), and resting respiratory rate (RR) across the life course (from age 2 y onward) in female and male patients. We analyzed cross-sectional retrospective data from two sources in parallel: from electronic health records (EHR) and from in-laboratory polysomnography (PSG) studies. The CO_2_ levels were measured as serum bicarbonate CO_2_ in the EHR data and as end-tidal CO_2_ in the PSG data. Our retrospective review of de-identified patient records from both sources (IRB #21-1984 for sleep data and #20-2103 for EHR data) was approved by the Institutional Review Board of the University of North Carolina at Chapel Hill.

### 2.2. Electronic Health Record (EHR)

We obtained the EHR data from the Carolina Data Warehouse at the University of North Carolina, Chapel Hill (UNC-CH); we requested the UNC-CH portion of the PCORnet (Patient-Centered Clinical Research Network) (http://www.pcornet.org/ accessed on 6 June 2025) data from the period 1 January 2018, to 31 December 2020, during the COVID years. The data set consisted of demographics, clinical observations, vitals, laboratory test results, and diagnoses with the Common Data Model (CDM) specification (version 5.1). All tables were linked through “encounters”—identifiers that are primarily used to connect information about the actual activities that occurred on any visit. We focused on four key variables of interest [oximetry O_2_ saturation (LOINC code: 59408-5), serum bicarbonate level (LOINC code: 2028-9), HR, and RR] together with age, sex, and BMI, and pre-processed the data set to have a single observed value from a single encounter for each key variable for each patient.

Features of the EHR data required some arbitrary choices in pre-processing the data for analysis. First, our EHRs included each patient’s year of birth but neither the year when the clinical encounter occurred nor the patient’s age at the encounter. Consequently, we estimated each patient’s age at the time of each encounter by using the formula: the maximum of either 0 or (2019 minus the patient’s birth year). We chose the year 2019 as a reference point because it is the middle year in our data range. Second, sometimes a key variable might have several distinct measured values recorded at the same encounter. When that occurred, we assigned the median of those values as the value of that variable at the encounter. Third, an individual patient might have multiple distinct encounters and have the same key variable measured at several of them; if so, we selected at random one among the patient’s several encounters where that variable was measured to provide the measured value for that variable for that patient. Fourth, an individual patient with multiple encounters might have measurements for only a subset of key variables spread over different encounters—never having every variable of interest measured at any single encounter or, perhaps, never having some key variables recorded at all. For such a patient, we randomly chose one encounter for each available key variable to provide a measured value for that patient for that key variable. This procedure resulted in one value per variable per patient; however, a given subject could possibly have values of different key variables coming from different encounters. In addition, this procedure resulted in different numbers of patients contributing values to each key variable in the EHR data.

For this study, we restricted attention to encounters coded as “AV” (ambulatory visit) or as “OV” (other ambulatory visit); 241,305 encounters involving 59,131 patients met that criterion. In addition, we restricted attention to encounters that provided a value for BMI, reducing the number of eligible encounters to 234,052 (97%) encounters involving 56,804 (96%) patients. Then, we removed encounters for patients under the age of 2 y and those with BMI below 15 or above 100 kg/m^2^. We considered outlying values to be measurements with O_2_ saturation above 100%, serum bicarbonate below 10 or above 50 mEq/L, heart rate below 40 or above 150 bpm, and respiratory rate below 5 or above 30 bpm. We excluded outlying values from subsequent consideration. We retained any encounters that had non-missing values for at least one of the four key variables (O_2_, bicarbonate, HR, and RR). After selecting one encounter per patient per key variable, the resulting EHR data set involved 53,252 distinct patients with values for least one key variable plus BMI—though fewer patients contributed data on any individual key variable: 23,336 for O_2_ saturation; 44,022 for serum bicarbonate; 22,595 for HR; and 18,496 for RR.

From the DIAGNOSIS table, we extracted major heart and lung related diagnoses based on ICD10 (International Classification of Diseases, Tenth Revision) codes: I25 for coronary heart disease (CHD), I26 for pulmonary embolism (PE), I50 for heart failure (HF), J43 for emphysema (EM), and J44 for chronic obstructive pulmonary disease (COPD). All diagnoses were made by the patient’s care provider and recorded in the medical record. At the randomly selected encounter used in our analyses, among the 53,252 patients contributing data, 5820 (10.9%) of them reported at least one of these five diagnoses (Supplemental Table S1).

### 2.3. In-Laboratory Polysomnography Study

We obtained de-identified records of patients referred by their healthcare providers for PSG studies at the sleep laboratory at UNC-CH between 1 January 2003, and 31 March 2024. This sleep laboratory is accredited by the American Academy of Sleep Medicine (AASM). Reasons for patient referral were not included in our data, though most were likely referred for evaluation of sleep apnea.

From PSG reports, we extracted basic demographic information such as sex, age (y), and body mass index (BMI) (kg/m^2^). We also extracted baseline (at the beginning of the PSG study) measures such as O_2_ saturation from finger oximetry, exhaled CO_2_ (in the form of end-tidal CO_2_ [ETCO_2_] from a BCI capnograph sampled through a nasal cannula), resting heart rate (HR), and resting respiratory rate. The PSG data were stored using a Stellate Systems polygraph (Montreal, QC, Canada) from 2003 to 2012, a Grass Systems polygraph (model 7D) from 2013 to 2018, and a Natus polygraph (Natus^®^ SleepWorks™ PSG Software, version: 9.0) after 2019.

We downloaded records for 43,744 PSGs representing 30,406 patients; we required information on BMI and eliminated 3,664 PSGs that were missing that information. Thus, 39,869 PSGs (91%) representing 28,120 patients (92%) had BMI information. We removed PSG records on patients under the age of 2 y and those with BMI below 15 kg/m^2^ or above 100 kg/m^2^. As with the EHR data, we considered outlying values to be measurements with O_2_ saturation above 100%, CO_2_ below 20 or above 60 mmHg, heart rate below 40 or above 150 bpm, and respiratory rate below 5 or above 30 bpm. We excluded PSGs containing outlying values for any of these variables from subsequent consideration. For patients with multiple PSG studies, we only kept their first PSG study. After these exclusions, 26,401 patients remained. We used only patients with complete data on all four key variables (HR, RR, O_2_, and CO_2_) plus BMI in the main analysis. The final PSG data set contained PSG studies for 21,477 patients (81% of those remaining after exclusions), with one PSG record per patient.

As with the EHR data, we extracted the presence of major heart- and lung-related diagnoses for these PSG patients: CHD, PE, HF, EM, and COPD. These diagnoses originated from the patient’s care provider and were downloaded to the sleep record from the original medical record. Among the 21,477 PSG patients contributing data, 2231 (10.4%) of them had at least one of these five diagnoses (Supplemental Table S1).

### 2.4. Statistical Analysis

Although we restricted our analysis of the PSG data to those patients with complete information on all four key variables (HR, RR, O_2_, and CO_2_), we did not apply this rule to the EHR data because only a small proportion of individuals had complete data for all four key variables in that data set (11,219 out of 53,252 or 21%).

We created 16 non-overlapping age groups: 2–5 y, 14 consecutive 5-year age groups starting at age 5 y, and 75 y or older. We summarized each quantitative variable for each sex separately using means and standard deviations (SD), and we used the Welch’s *t*-test to compare distributions between women and men. For categorical variables, we report proportions and used Pearson’s chi-squared test to compare between women and men.

For each of the key variables (O_2_, CO_2_/bicarbonate, HR, RR), we used general linear regression models with sex, age group, and sex by age group interactions as predictors to estimate separate age trajectories for each sex. For the PSG data, the regression models also included year of study as a categorical variable to account for changes in technology over time. In a separate set of analyses for both the PSG and EHR data sets, we also adjusted for BMI by including 3-degree-of-freedoom cubic spline for BMI and adjusted for disease status by including an indicator variable for each of five major diseases that may affect gas exchange (CHD, PE, HF, EM, and COPD). We compared female–male differences separately in each age group and used the Benjamini–Hochberg procedure to adjust *p*-values for multiple testing.

We also constructed contrasts among age- and sex-specific mean values to investigate whether male–female difference in mean response for the key variables changed from before to after puberty in juveniles and from before to after perimenopause in adults. For the before-to-after puberty comparison, we constructed the post-minus-pre difference by taking the difference between juveniles at least 10 years old but not older than 20 years and children from 2 up to 10 years old in males and females separately. We then subtract the difference in males from that in females. For the before-to-after perimenopause comparison, we constructed the post-minus-pre difference by subtracting young adults at least 20 but younger than 45 years old (≥20 and <45 years) from adults 55 years and older (≥55) in males and females separately. We then subtract the difference in males from that in females. Positive values for these contrasts indicate a larger positive female-minus-male difference in the older age group. If progesterone enhances gas exchange, we expect a larger female–male difference for O_2_ and CO_2_ in older post-pubertal patients compared with younger pre-pubertal juveniles and, conversely, a smaller female–male difference in older adults than in younger adults—though the sign of the difference may depend on whether levels of the outcome are higher in women or in men. All analyses were performed used R (version 4.3.0).

## 3. Results

### 3.1. Subject Characteristics

#### 3.1.1. EHR Data

Our EHR data set contained a total of 53,252 unique patients, among whom 32,760 (61.5%) were female and 20,492 (38.5%) were male (Table 1). Although age group distributions differed in detail between females and males (Table 1), for both sexes, older age groups were more prevalent than younger ones. The average ages were 54 and 52 years old for females and males, respectively (Table 2). On average, females (31 kg/m^2^) had a higher BMI than males (29 kg/m^2^). The average levels of O_2_ saturation were similar between the sexes (females, 97.3%; males, 97.0%), as were the average serum bicarbonate levels (females, 26.6 mEq/L; males, 26.9 mEq/L). The average heart rates of females and males were 82 bpm and 80 bpm, respectively. The average respiratory rates for both sexes were about 17 bpm. Higher proportions of males than females had been diagnosed with CHD (Table 2).

#### 3.1.2. PSG Data

Our PSG data set included 21,477 PSG studies, each from a distinct patient, among whom 10,568 (49.2%) were females and 10,909 (50.8%) were males (Table 1). The age group distributions differed in detail between females and males (Table 1) but, for both sexes, older age groups were more prevalent than younger ones—though this tendency was less pronounced than in the EHR data set. The average ages were 45 and 44 years old for females and males, respectively (Table 2). Females (33 kg/m^2^) had, on average, a higher BMI than males (31 kg/m^2^). The average levels of O_2_ saturation were similar between the sexes (females, 96.7%; males, 96.3%), as were the end-tidal CO_2_ levels (females, 40 mmHg; males, 39.7 mmHg). The average heart rate of females (76 bpm) was about 2 bpm higher than that in males (74 bpm). The average respiratory rates for both sexes were around 16 bpm. Except for CHD and COPD, similar proportions of each sex were diagnosed with other major cardiovascular diseases (Table 2). CAD was more frequently diagnosed among males in the PSG data set whereas COPD was more frequently diagnosed among females.

### 3.2. Age Trajectories of Key Variables

#### 3.2.1. Pulse Oximetry Oxygen Saturation

**Figure 1:**
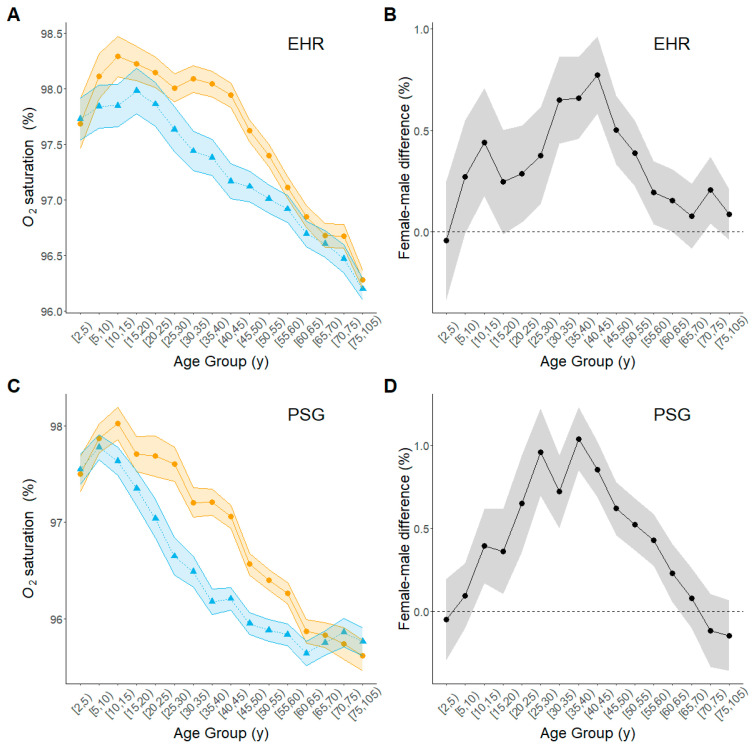
Estimated age trajectories of O_2_ saturation for men and women across the life course. Shaded bands outline 95% upper and lower confidence limits for the estimate at each age group. Amber circles, women; blue triangles, men; black circles, female-minus-male difference. (**A**) Estimated trajectories of mean O_2_ saturation from the EHR data set; (**B**) age-group-specific difference in mean O_2_ saturation from the EHR data set; (**C**) estimated trajectories of mean O_2_ saturation from the PSG data set; (**D**) age-group-specific difference in mean O_2_ saturation from the PSG data set.

In both the EHR and PSG data, the mean O_2_ saturation levels generally declined with increasing age, though with some evidence of an upward trend at the youngest ages, and women had comparable or higher mean O_2_ saturation levels than men throughout life (Figure 1A,C, Supplemental Tables S2 and S3). O_2_ saturation levels were similar in both sexes before age 20 y and after age 60 y but diverged during early adulthood. In fact, the mean female–male difference in O_2_ saturation increased until middle age and then declined in later life (Figure 1). The largest sex difference in mean O_2_ saturation in the EHR data was for the 40–45 y age group (mean = 0.80, 95% CI: 0.61, 1.99, *p* < 1 × 10^−5^. In the PSG data, the largest sex difference was in the 35–40 y age group (mean = 1.04, 95% CI: 0.85, 1.23, *p* < 1 × 10^−5^). Overall, the O_2_ saturation was slightly higher in the EHR data compared with the PSG data across the age groups, reflecting the population difference. The models that adjusted these estimates for BMI did not modify these reported patterns (Supplemental Figure S1).

#### 3.2.2. Serum Bicarbonate Level (EHR Data)

**Figure 2:**
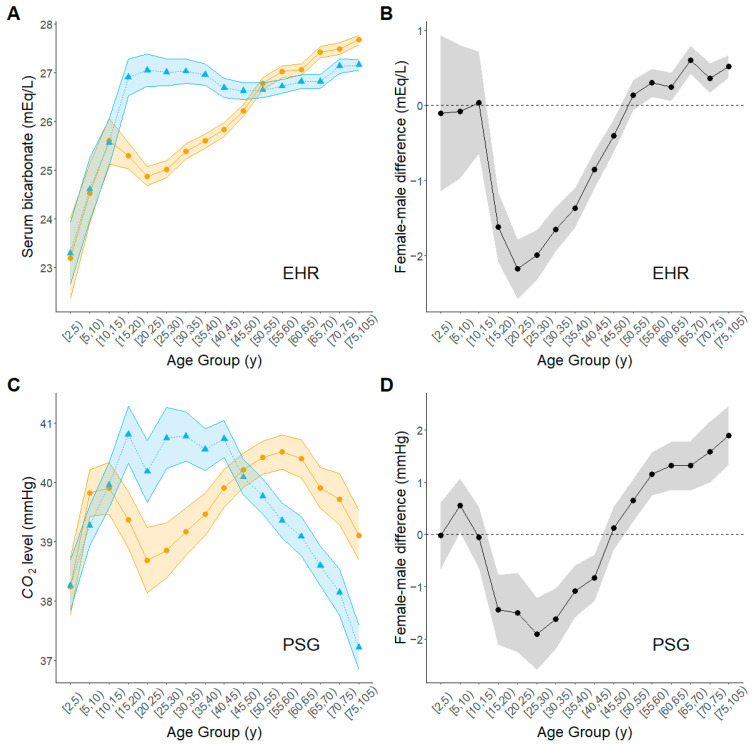
Estimated age trajectories of CO_2_ saturation for men and women across the life course. Amber circles, women; blue triangles, men; black circles, female-minus-male difference. (**A**) Estimated trajectory in serum bicarbonate from EHR data set; (**B**) Female-male difference from EHR data set; (**C**) Estimated trajectory in end-tidal CO_2_ from PSG data set; (**D**) Female-male difference from PSG data set. Plotted points represent estimates from fitted regression models for each age group; shaded bands outline 95% upper and lower confidence limits for the estimate at each age group.

The mean serum bicarbonate levels were comparable for women and men before age 15 y, then diverged with the mean level in women staying below that in men until about age 55 y. After that, the mean levels in women were higher than those in men (Figure 2A,B, Supplemental Table S4). The mean serum bicarbonate levels increased in both sexes up to the 10–15 y age group; after that their trajectories diverged. In women, mean levels declined from 25.5 mEq/L in the 10–15 y age group to around 24.8 mEq/L in the 20–25 y age group before resuming a steady increase that continued through the oldest age groups. In men, the initial increase continued until the 20–25 y age group reaching a mean level of 27 mEq/L. After that, the mean serum bicarbonate levels in men remained reasonably stable, decreasing slightly then increasing, through the oldest age groups. The largest mean female-minus-male difference in serum bicarbonate level was −2.23 mEq/L (mean = −2.23, 95% CI: −2.62, −1.83, *p* < 1 × 10^−5^) in the 20–25 y age group (women lower than men). The pattern was similar with estimates from the models adjusted for BMI (Supplemental Figure S2).

#### 3.2.3. End-Tidal CO_2_ (PSG Data)

The age trajectories of the mean levels of end-tidal CO_2_ for women and men in the PSG data (Figure 2C,D, Supplemental Table S5) exhibited broadly similar patterns to those of the mean serum bicarbonate levels in the EHR data (Figure 2A,B). The mean end-tidal CO_2_ levels were comparable for women and men before age 15 y, then diverged with the mean level in women staying below that in men until about age 45 y. After age 50 y, mean levels in women exceeded those in men. The mean end-tidal CO_2_ levels increased rapidly with age in both women and men before the 10–15 y age group. In women, levels then dropped until the 20–25 y age group, followed by a steady increase until the 55–60 y age group, and further followed by another drop through the oldest age groups. In men, mean levels of end-tidal CO_2_ continued to increase until the 15–20 y age group, then leveled off through the 40–45 y age group, and declined linearly from that age onward. Between age groups 15–20 y and 40–45 y, the mean levels of end-tidal CO_2_ were lower in women than in men, but that ordering reversed beginning with the 50–55 y age group. Including adjustments for BMI did not alter these general patterns (Supplemental Figure S2).

Although the age trajectories for serum bicarbonate in the EHR data and for end-tidal CO_2_ in the PSG data were broadly similar, we noted one clear difference in the older age groups. After about age 55 y, the mean serum bicarbonate levels gradually increased through the oldest ages in both women and men in the EHR data, whereas, during that same period, mean end-tidal CO_2_ levels in the PSG data fell sharply (Figure 2).

#### 3.2.4. Heart Rate

**Figure 3:**
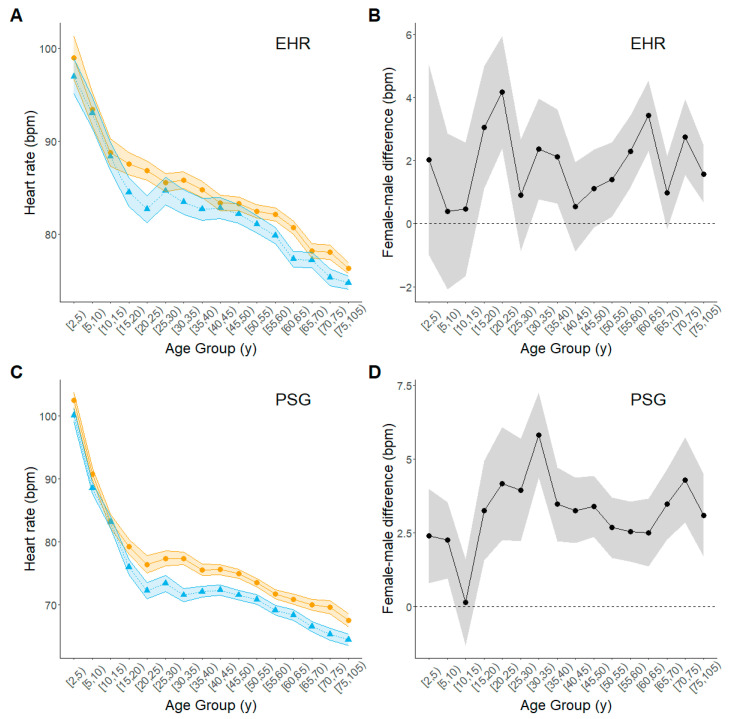
Estimated age trajectories of heart rate for men and women across the life course. Amber circles, women; blue triangles, men; black circles, female-minus-male difference. (**A**) Estimated trajectory from EHR data set; (**B**) Female-male difference from EHR data set; (**C**) Estimated trajectory from PSG data set; (**D**) Female-male difference from PSG data set. Plotted points represent estimates from fitted regression models for each age group; shaded bands outline 95% upper and lower confidence limits for the estimate at each age group.3.2.5. Respiratory Rate.

In both the EHR and PSG data sets, and for both sexes, mean HR exhibited similar age trajectories across the life course (Figure 3; Supplemental Tables S6 and S7). Mean HR decreased relatively rapidly with age up to about age 20 or 25 years; at older ages, it continued to decrease but at a slower rate until the oldest ages. In general, especially in the PSG data, women had slightly higher mean HR than men after the rate of decrease slowed. Before that, boys and girls had more similar mean HRs in both data sets. Patterns were similar after adjustment for BMI (Supplemental Figure S3).

**Figure 4:**
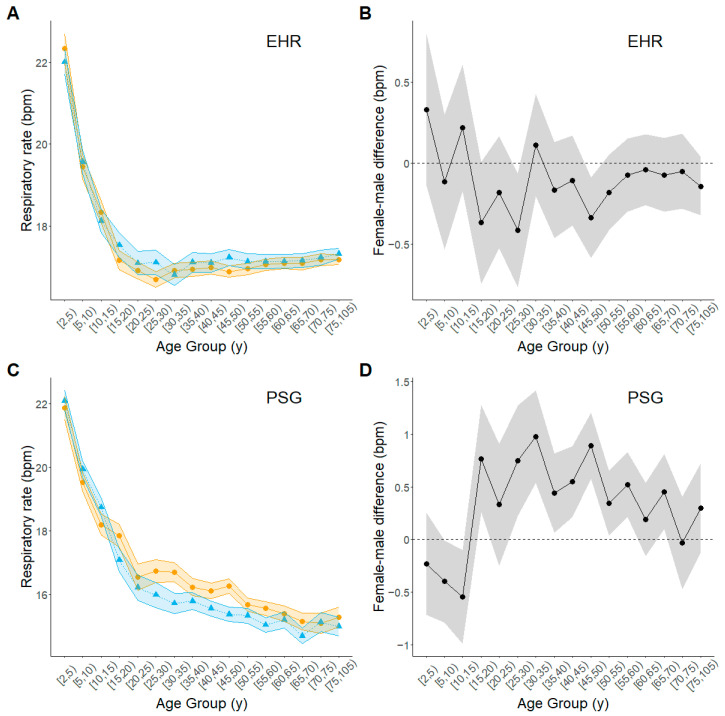
Estimated age trajectories of respiratory rate for men and women across the life course. Amber circles, women; blue triangles, men; black circles, female-minus-male difference. (**A**) Estimated trajectory from EHR data set; (**B**) Female-male difference from EHR data set; (**C**) Estimated trajectory from PSG data set; (**D**) Female-male difference from PSG data set. Plotted points represent estimates from fitted regression models for each age group; shaded bands outline 95% upper and lower confidence limits for the estimate at each age group.

The age trajectories of mean RR were broadly similar to those of mean HR but differed in some details (Figure 4, Supplemental Tables S8 and S9). In both data sets and for both sexes, mean RR decreased rapidly with increasing age until about age 20 or 25 y. After that, RR remained relatively unchanged at about 16–17 breathes per minute for both men and women in the EHR data but continued to decline gradually in the PSG data. In the EHR data, women and men had about the same mean RR throughout the life course; whereas, in the PSG data, women had a slightly higher mean RR than men after age 15 y, though mean RR for both sexes was nearly the same in the oldest age groups. After adjustment for BMI, any differences between women and mean in mean RR were attenuated (Supplemental Figure S4).

#### 3.2.5. Evidence Supporting the Role of Progesterone in Gas Exchange

We used the estimated age trajectories to examine a possible association of progesterone with the four key outcome variables. We reasoned that if progesterone is associated with an outcome, the association would manifest by the female-minus-male mean difference in an outcome changing from before to after an age corresponding to puberty in juveniles and before to after an age corresponding to menopause in adults. Because we lack measured progesterone or dates of menarche or menopause for our subjects, we used the age groups described in the Methods to define pre- and post-pubertal and pre- and post-menopausal periods (we omitted some age groups as peri-menopausal).

We observed a positive difference in female-minus-male differences in O_2_ saturation between post-pubertal and pre-pubertal age groups and, correspondingly, a negative difference in female-minus-male differences between post-menopausal and pre-menopausal age groups in both the EHR and PSG datasets (Table 3). Likewise, we found a negative difference in female-minus-male differences in both end-tidal CO_2_ and serum bicarbonate levels between post-pubertal and pre-pubertal age groups and a positive difference between post-menopausal and pre-menopausal age groups (Table 3). These results support the notion that progesterone appears to be associated with increased O_2_ saturation and decreased CO_2_ levels in women between menarche and menopause. Few sex differences in both respiratory rate and heart rate between post-menopausal and pre-menopausal and between post-pubertal and pre-pubertal comparisons were statistically significant.

## 4. Discussion

Using cross-sectional data from two sources, we characterized the mean levels of O_2_ saturation, CO_2_, HR, and RR across the life course for women and men. The subjects from the two data sources, EHR data from ambulatory visits to UNC hospitals and data from patients referred for PSG studies to the UNC Sleep Clinic, differed in age distribution and other characteristics. The age groups from 15 to 30 y had proportionally fewer participants in the PSG data than in the EHR data, in accord with expected referral patterns. In the EHRs, CO_2_ levels were recorded as serum bicarbonate; whereas, in the PSG records, CO_2_ levels were recorded as end-tidal CO_2_. Despite such differences, age trajectories from both data sets were reasonably similar across the life course for all four outcomes.

In both data sets, women in the ages of 15–45 y had significantly lower serum bicarbonate level and end-tidal CO_2_ level and higher O_2_ saturation than men of similar ages. That age range approximately mirrors the time between menarche and menopause. Progesterone level rises in menarche girls and falls in postmenopausal women. Increases in progesterone concentrations are seen in girls age 9–15 y [42,43,44]. After menopause, the depletion of ovarian follicles leads to permanent reductions in serum progesterone level to a low of <0.2–0.5 ng/mL compared with about 5–20 ng/mL during the follicular phase of the menstrual cycle [45]. We speculate that sex hormones, especially progesterone, play an important role in driving the sex differences in mean O_2_ and CO_2_ levels throughout life. Progesterone is a known strong stimulator of respiration [21,22,23,24,25]; it has powerful vasodilatory and natriuretic effects [46,47]. Premenopausal women are at lower risk of developing hypertension and coronary heart disease than men of the same age and the cardiovascular risk increases only after the cessation of ovarian function [48]. Besides hormonal effects, differences in upper airway structure and the regulatory control of airway muscles may contribute to the sex differences [49,50,51].

Among those over age 60 y, although women and men had similar levels of O_2_ saturation in both data sets, women had higher serum bicarbonate in the EHR data and higher end-tidal CO_2_ in the PSG data than men of similar ages. The trajectories of both serum bicarbonate level and end-tidal CO_2_ crossed around age 55–60 years, suggesting that, among those older than 55 yr, women may have decreased gas exchange capacity compared with men. Perhaps, respiratory needs are lower in older women; on the other hand, older women may have reduced capacity for gas exchange at a physiological level. The second possibility seems more likely. An elevated serum bicarbonate level in the blood may result from either a metabolic alkalosis or as compensation to respiratory acidosis [52]. A normal blood serum bicarbonate level is between 22 and 29 mEq/L. A mean level of ~28 mEq/L in elderly women remains within the normal range but towards its upper limit. In fact, our data suggest that relatively low O_2_ saturation, relatively high serum bicarbonate level (total blood CO_2_), and relatively high end-tidal CO_2_ in elderly women compared with elderly men may be associated with increased prevalence of sleep disordered breathing. This possibility is consistent with the finding that the prevalence of obstructive sleep apnea in elderly women continued to increase with age [53]. Elderly women with disordered breathing had an increased risk of developing cognitive impairment [54]. Increased awareness by clinicians to this population of patients could improve patients’ care.

The age trajectory of end-tidal CO_2_ level in women showed a cyclic pattern in our PSG data. In the EHR data, the pattern of serum bicarbonate level with age in women partly mimicked that cyclic pattern by exhibiting a rapid rise, then a fall followed by another rise again. The obvious difference between those two age trajectories was that end-tidal CO_2_ decreased with age after age 55 y; whereas serum bicarbonate continued to increase. Women at the ages of 20–25 y had high O_2_ saturation and low serum bicarbonate or low end-tidal CO_2_, suggesting that women in that age range have the best performance of gas exchange.

Aging is associated with physiological changes in respiratory function; it tends to diminish the reserve of the respiratory system in cases of acute disease [27]. Gas exchange in elderly subjects is well preserved at rest, however, and can adapt to high levels of exercise with regular training. It is the ability to deal with pathological conditions such as infection or heart failure that tend to diminish with aging [27].

### 4.1. Limitations and Strengths

Our analysis has several limitations. First, our data came from a tertiary hospital population, not a designed sample from the general population in a well-defined geographical area. This means that our findings may not generalize to the broader population. Ideally, we would like our samples to be representative of the population in a region surrounding the UNC hospital; however, both the EHR and PSG data may not accurately represent such a general population because of selection bias—patients who use physicians affiliated with the UNC system or who are referred to the UNC sleep lab may differ in systematic ways from the local population. The PSG patients likely skew towards being less healthy than the general public and also less healthy than the patients in the EHR data. The PSG patients were referred to the sleep lab for the evaluation of suspected sleep disordered breathing, whereas many EHR patients underwent routine physical exams. The PSG patients tended to be more obese than the EHR patients. The differences, e.g., respiratory and heart rates, between the two datasets may be directly related to differential selection bias between the two samples.

A second limitation, only applicable to the EHR data, is that, because we did not know the exact date of each encounter, we had to approximate each patient’s age; our approximation rule provided estimates within ±1 y of the patient’s actual age. This approximation means that some patients were misclassified into a 5-y interval adjacent to the interval they belonged in. This misclassification will induce some bias in the sex-specific estimated means and may tend to reduce apparent changes between adjacent intervals. Nevertheless, because we expect the probability of misclassification to be the same for women and men in any age interval, the female–male differences in age-interval-specific means should experience little bias due to age misclassification.

Third, our data are cross-sectional, not longitudinal, so that the age-related trends that we saw may imperfectly reflect the trajectories that an individual subject would experience with aging. Fourth, we do not have measured progesterone levels for the patients to further test the hypothesis that the sex differences we observed are related to progesterone; instead, we relied on age as a proxy for developmental events, like puberty or menopause, that signal hormonal changes. Fifth, we lacked data to explicitly consider race in our analyses, but race (or skin color) has an effect on the ability of external devices to accurately detect blood oxygen saturation levels through the skin [55]. Nevertheless, because our primary comparisons are between sexes, we believe that skin color is unlikely to change the observed patterns.

The principal strength of our study is the parallel analyses using two different data sets. Both data sets were large, containing both urban and rural subjects who were presumed ethnically diverse. That the shapes of the age trajectories and age-related change in the female–male differences from both data sets were strikingly similar offers reassurance that our results are applicable more broadly, despite the limitations mentioned earlier.

### 4.2. Perspectives and Significance

Sex differences in physiology and diseases are widely recognized. Sex hormones play an important role in these differences. Numerous studies have demonstrated that premenopausal women may be protected from cardiovascular diseases compared with postmenopausal women. Premenopausal and postmenopausal women also experience differences in sleep quality and sleep disorders. Our results of significant differences in both the gas exchange components (O_2_ saturation and CO_2_ levels) between menarche and menopause support the established role of sex hormones in respiratory function. Our data also demonstrate that special attention needs to be paid to elderly women in areas such as sleep-disordered breathing and cardiovascular health, as their oxygen saturation declines faster with age and their CO_2_ levels remain higher than those of men of similar ages. This worsening trend in elderly women appears to continue with age.

## 5. Conclusions

The trajectories of oximetry O_2_ saturation across the lifespan in both the EHR and PSG datasets were notably comparable. Similarly, the trajectory of end-tidal CO_2_ in the PSG data closely mirrored that of serum bicarbonate levels from the EHR. Throughout most of their lives, the women exhibited higher O_2_ saturation than the men. This difference diminished in elderly individuals, however. The women between menarche and menopause had significantly lower levels of end-tidal CO_2_ and serum bicarbonate compared with men of the same age group. Post menopause, women showed higher levels in both end-tidal CO_2_ and serum bicarbonate levels. These findings further underscore the influence of progesterone on gas exchange.

## Figures and Tables

**Table 1:** Age group distributions of females and males between EHR data set and PSG data set.

Age Group (y)	EHR Data Set (N = 53,252)				PSG Data Set (N = 21,477)			
	Female (N = 32,760)		Male (N = 20,492)		Female (N = 10,568)		Male (N = 10,909)	
	N	%	N	%	N	%	N	%
[2,5)	276	0.8	396	1.9	376	3.6	510	4.7
[5,10)	361	1.1	387	1.9	583	5.5	771	7.1
[10,15)	460	1.4	427	2.1	456	4.3	586	5.4
[15,20)	852	2.6	491	2.4	393	3.7	386	3.5
[20,25)	1310	4.0	541	2.6	285	2.7	323	3.0
[25,30)	1603	4.9	657	3.2	411	3.9	335	3.1
[30,35)	1951	6.0	830	4.1	556	5.3	507	4.6
[35,40)	2062	6.3	1002	4.9	694	6.6	730	6.7
[40,45)	2338	7.1	1166	5.7	886	8.4	923	8.5
[45,50)	2775	8.5	1473	7.2	1070	10.1	986	9.0
[50,55)	2884	8.8	1761	8.6	1151	10.9	982	9.0
[55,60)	3063	9.3	2047	10.0	1090	10.3	1032	9.5
[60,65)	3062	9.3	2137	10.4	859	8.1	808	7.4
[65,70)	2842	8.7	2117	10.3	758	7.2	821	7.5
[70,75)	2595	7.9	1828	8.9	485	4.6	583	5.3
[75,105)	4326	13.2	3232	15.8	515	4.9	626	5.7

**Table 2:** Key characteristics of females and males in the EHR data set and the PSG data set.

		EHR Data Set ^a^					PSG Data Set ^b^			
					Female–Male Difference ^c^				Female–Male Difference ^c^	
Outcome	Sex	Total Patients	Mean	SD	Test Statistic	*p* Value	Mean	SD	Test Statistic	*p* Value
Age (y)	F	32,760	52	20			45	21		
	M	20,492	54	21	−9.92	<0.0001	44	22	3.52	0.01
BMI (kg/m^2^)	F	32,760	31	8			33	11		
	M	20,492	29	7	17.91	<0.0001	51	9	20	<0.0001
O_2_ saturation (%)	F	14,132	97.37	1.84			96.73	2.04		
	M	9204	96.98	1.9	15.64	<0.0001	96.33	2.01	14.36	<0.0001
CO_2_ ^d^	F	27,294	26.6	3.2			40	5		
	M	16,628	26.8	3.2	−6.76	<0.0001	39.7	4.9	3.89	<0.0001
HR (bpm)	F	13,724	82	13			76	14		
	M	8871	80	14	11.09	<0.0001	74	15	12.81	<0.0001
RR (bpm)	F	11,217	17.18	2.34			16.4	3.9		
	M	7279	17.44	2.51	−6.37	<0.0001	16.2	4.1	3.82	<0.0001
**Disease**	**Sex**	**Total Patients**	**N**	**%**	**Test Statistic**	***p* Value**	**N**	**%**	**Test Statistic**	***p* Value**
Coronary Heart Disease	F	32,760	1120	3.4			374	3.5		<0.0001
	M	20,492	1792	8.7	691.8	<0.0001	665	6.1	76.23	
Pulmonary Embolism	F	32,760	73	0.2			99	0.9		0.30
	M	20,492	76	0.4	9.9	0.002	88	0.8	1.05	
Emphysema	F	32,760	1145	3.5			248	2.3		0.40
	M	20,492	1139	5.6	130.7	<0.0001	236	2.2	0.82	
Heart Failure	F	32,760	247	0.8			35	0.3		0.70
	M	20,492	191	0.9	4.9	0.027	40	0.4	0.19	
COPD	F	32,760	926	2.8			468	4.4		0.002
	M	20,492	722	3.5	20.4	<0.0001	391	3.6	9.96	

^a^ In the EHR data set, all patients contributed data on disease status, age, and BMI but variable numbers contributed data on the remaining characteristics. ^b^ In the PSG data set, all patients contributed data on all characteristics. The total numbers of females and males were 10,568 and 10,909, respectively, for all variables in the table. ^c^ Test statistics and *p* values are from Welch’s two-sample t-statistic for continuous characteristics and from Pearson’s chi-squared statistic for disease comparisons. ^d^ Measured as serum bicarbonate (mEq/L) in EHR patients and as end-tidal CO_2_ (mmHg) in the PSG patients.

**Table 3:** Comparisons of outcome changes from before to after puberty and from before to after menopause.

		Female			Male			Female−Male			
Outcome	Pre–Post Boundary	Estimate ^a^	Confidence Limits		Estimate	Confidence Limits		Estimate	Confidence Limits		*p* Value
			Lower	Upper		Lower	Upper		Lower	Upper	
EHR											
O_2_	Puberty	0.37	0.18	0.56	0.17	−0.02	0.37	0.20	−0.07	0.47	0.15
	Menopause	−1.31	−1.38	−1.24	−0.94	−1.03	−0.85	−0.37	−0.48	−0.25	<0.0001
HCO_3_^−^	Puberty	1.65	1.07	2.23	2.22	1.67	2.76	−0.57	−1.37	0.22	0.16
	Menopause	1.98	1.88	2.07	0.05	−0.09	0.18	1.93	1.77	2.09	<0.0001
HR	Puberty	−8.42	−10.19	−6.65	−9.16	−10.82	−7.50	0.74	−1.69	3.16	0.61
	Menopause	−6.12	−6.64	−5.59	−6.58	−7.29	−5.87	0.46	−0.42	1.34	0.61
RR	Puberty	−3.10	−3.39	−2.80	−3.07	−3.37	−2.78	−0.02	−0.44	0.39	0.91
	Menopause	0.27	0.17	0.37	0.17	0.04	0.31	0.10	−0.08	0.27	0.55
**PSG**											
O_2_	Puberty	0.18	0.01	0.35	−0.17	−0.33	−0.02	0.35	0.12	0.59	0.0027
	Menopause	−1.49	−1.59	−1.40	−0.74	−0.84	−0.65	−0.75	−0.88	−0.61	<0.0001
ETCO_2_	Puberty	0.60	0.16	1.05	1.61	1.21	2.02	−1.01	−1.61	−0.40	0.0011
	Menopause	0.71	0.46	0.96	−2.12	−2.36	−1.87	2.83	2.48	3.18	<0.0001
HR	Puberty	−15.35	−16.47	−14.23	−14.73	−15.75	−13.71	−0.62	−2.14	0.89	0.42
	Menopause	−6.50	−7.12	−5.87	−5.55	−6.16	−4.93	−0.95	−1.83	−0.07	0.07
RR	Puberty	−2.68	−3.02	−2.34	−3.1	−3.41	−2.79	0.42	−0.03	0.88	0.07
	Menopause	−1.16	−1.35	−0.97	−0.84	−1.03	−0.65	−0.32	−0.59	−0.06	0.04

^a^ The estimate is a difference of differences in average outcome, namely, the post-minus-pre puberty (or menopause) difference in the female mean subtracted from the same post-minus-pre difference in the male mean.

## Data Availability

The original contributions presented in this study are included in the article. Further inquiries can be directed to the corresponding author.

## Supplementary Materials

The following supporting information can be downloaded at: https://www.mdpi.com/article/10.3390/biom15060884/s1, Figure S1: Estimated age trajectories of O_2_ saturation for men and women across the life course after adjustment for BMI; Figure S2: Estimated age trajectories of CO_2_ levels for men and women across the life course after adjustment for BMI; Figure S3: Estimated age trajectories of heart rate for men and women across the life course after adjustment for BMI; Figure S4. Estimated age trajectories of respiratory rate for men and women across the life course after adjustment for BMI; Table S1. Counts for the presence (1) and absence (0) of five relevant diseases in the EHR and PSG data; Table S2. Age-group-specific estimates and 95% confidence limits for trajectories of O_2_ saturation (%) in the EHR data set (N = 23,336) for females and males; Table S3. Age-group-specific estimates and 95% confidence limits for trajectories of O_2_ saturation (%) in the PSG data set (N = 21,477) for females and males; Table S4. Age-group-specific estimates and 95% confidence limits for trajectories of serum bicarbonate level (mEq/L) in the EHR data set (N = 44,202) for females and males; Table S5. Age-group-specific estimates and 95% confidence limits for trajectories of end-tidal CO_2_ level (mmHg) in the PSG data set (N = 21,477) for females and males; Table S6. Age-group-specific estimates and 95% confidence limits for trajectories of heart rate (bpm) in the EHR data set (N = 22,595) for females and males; Table S7. Age-group-specific estimates and 95% confidence limits for trajectories of heart rate (bpm) in the PSG data set (N = 21,477) for females and males; Table S8. Age-group-specific estimates and 95% confidence limits for trajectories of respiratory rate (bpm) in the EHR data set (N = 18,496) for females and males; Table S9. Age-group-specific estimates and 95% confidence limits for trajectories of respiratory rate (bpm) in the PSG data set (N = 21,477) for females and males.

## Abbreviations

The following abbreviations are used in this manuscript:

BMI	Body mass index
CHD	Coronary heart disease
COPD	Chronic obstructive pulmonary disease
EHR	Electronic health record
EM	Emphysema
HF	Heart failure
PE	Pulmonary embolism
PSG	Polysomnography
RR	Respiratory rate
