# Supplementary material for: Age Trajectories of O2 Saturation and Levels of Serum Bicarbonate or End-Tidal CO2 Across the Life Course of Women and Men: Insights from EHR and PSG Data
Source: Biomolecules. 2025 Jun 17;15(6):884. doi: 10.3390/biom15060884 (PMC12190716; doi:10.3390/biom15060884)
Supplement: Supplementary file 1 [file biomolecules-15-00884-s001.zip › biomolecules-3578045-supplementary.pdf]

Supplementary Figures and Tables for

Age trajectories of O_2 saturation and levels of serum bicarbonate or end-tidal CO_2 across the life course of women and men: Insights from EHR and PSG Data

by Leping Li, Min Shi, David M. Umbach and Zheng Fan

Supplementary Figure S1. Estimated age trajectories of O_2 saturation for men and women across the life course after adjustment for BMI and disease status. Plotted points represent estimates from fitted regression models for each age group; shaded bands outline 95% upper and lower confidence limits for the estimate at each age group. Amber circles, women; blue triangles, men; black circles, female-minus-male difference. Panels: (**A**). Estimated trajectories of mean O_2 saturation for men and women from the EHR data set; (**B**). Age-group-specific difference in mean O_2 saturation between women and men from the EHR data set; (**C**). Estimated trajectories of mean O_2 saturation for men and women from the PSG data set; (**D**). Age-group-specific difference in mean O_2 saturation between women and men from the PSG data set.

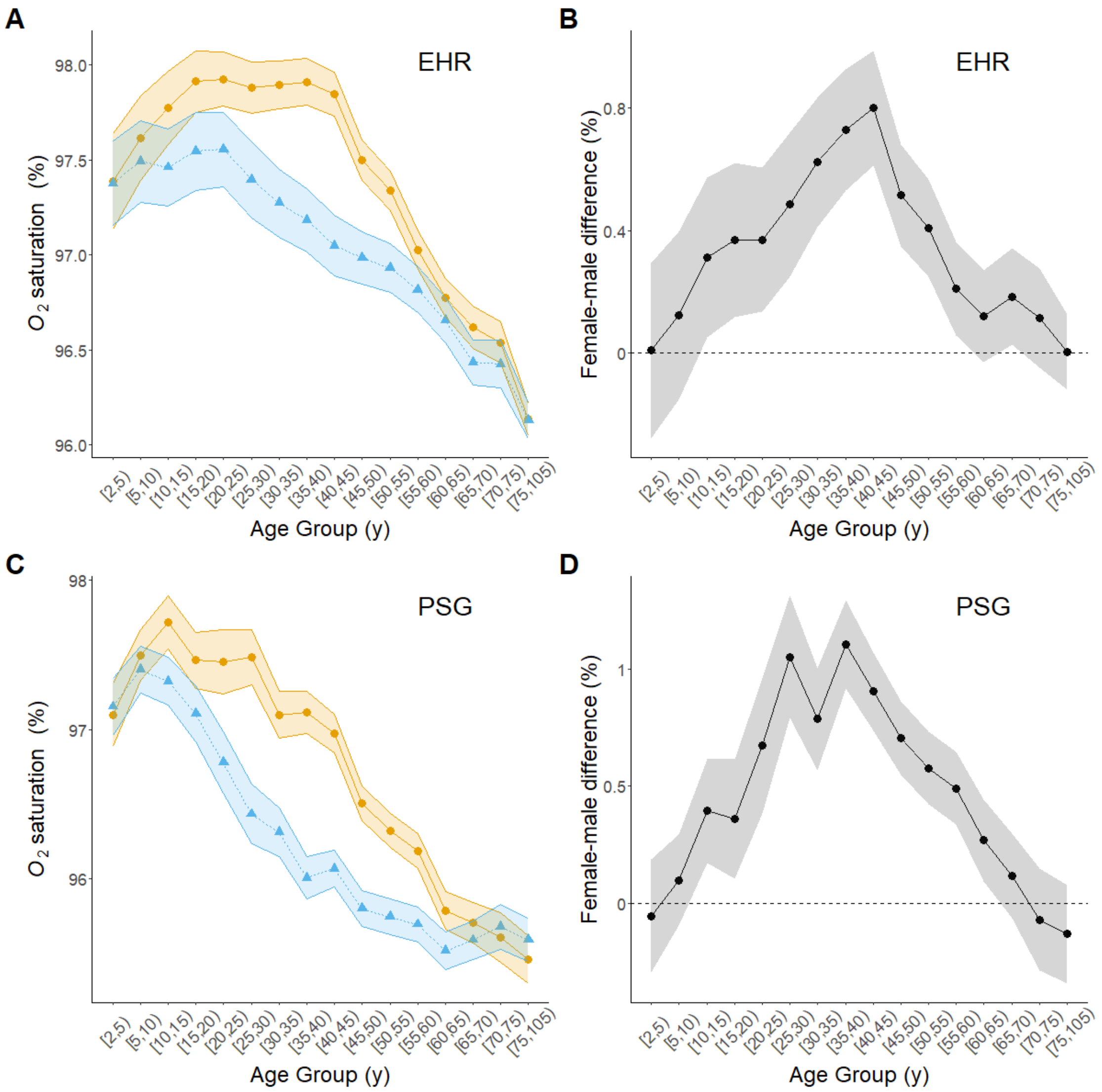

Supplementary Figure S2. Estimated age trajectories of CO_2 levels for men and women across the life course after adjustment for BMI and disease status. Plotted points represent estimates from fitted regression models for each age group; shaded bands outline 95% upper and lower confidence limits for the estimate at each age group. Amber circles, women; blue triangles, men; black circles, female-minus-male difference. Panels: (**A**). Estimated trajectories of mean serum bicarbonate for men and women from the EHR data set; (**B**). Age-group-specific differences in mean serum bicarbonate level between women and men from the EHR data set; (**C**). Estimated trajectories of mean end-tidal CO_2 for men and women from the PSG data set; (**D**). Age-group-

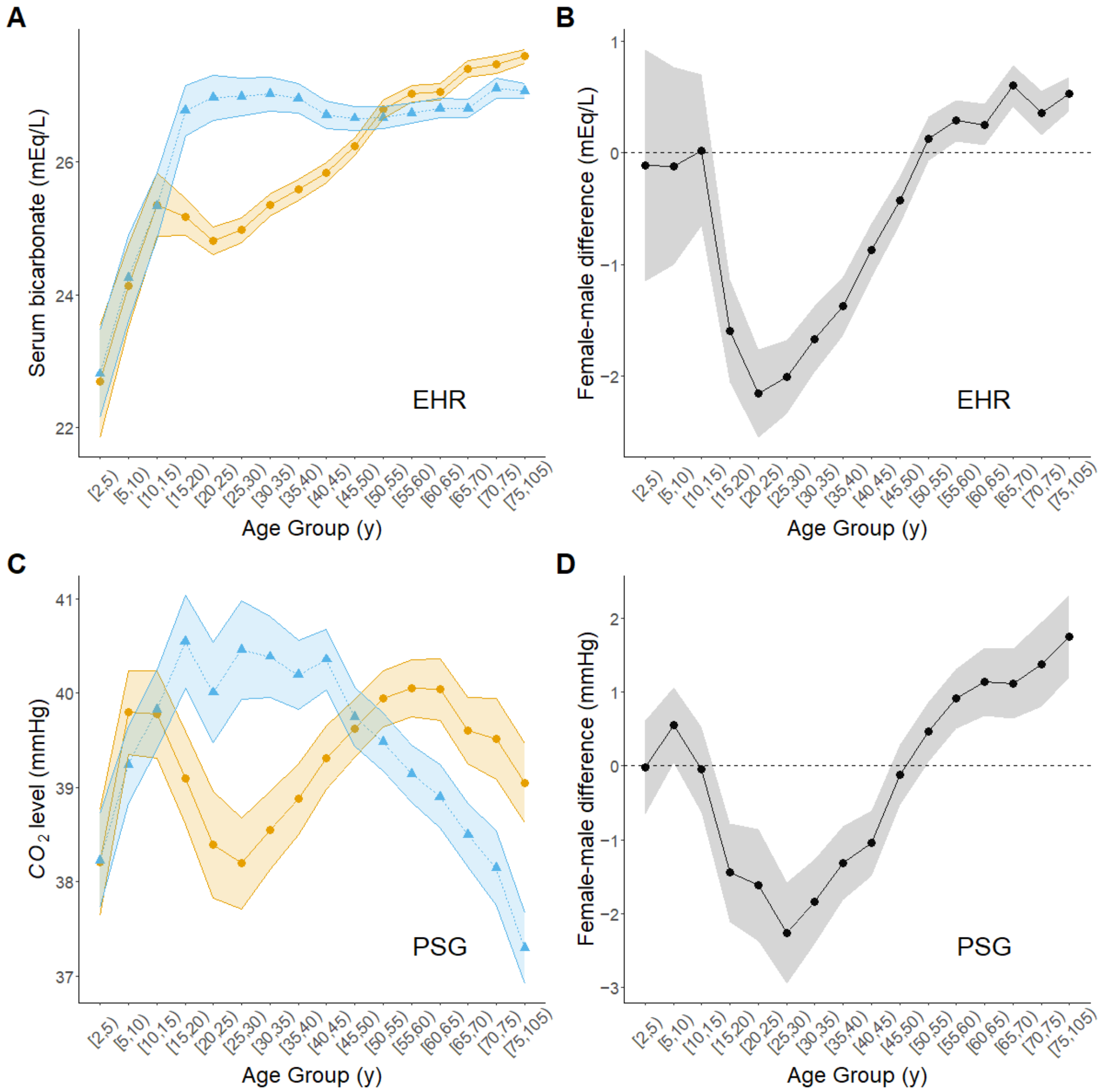

specific differences in end-tidal CO_2 between women and men from the PSG data set.

Supplementary Figure S3. Estimated age trajectories of heart rate for men and women across the life course after adjustment for BMI and disease status. Plotted points represent estimates from fitted regression models for each age group; shaded bands outline 95% upper and lower confidence limits for the estimate at each age group. Amber circles, women; blue triangles, men; black circles, female-minus-male difference. Panels: (**A**). Estimated trajectories of mean heart rate for men and women from the EHR data set; (**B**). Age-group-specific differences in mean heart rate between women and men from EHR data set; (**C**). Estimated trajectories of mean

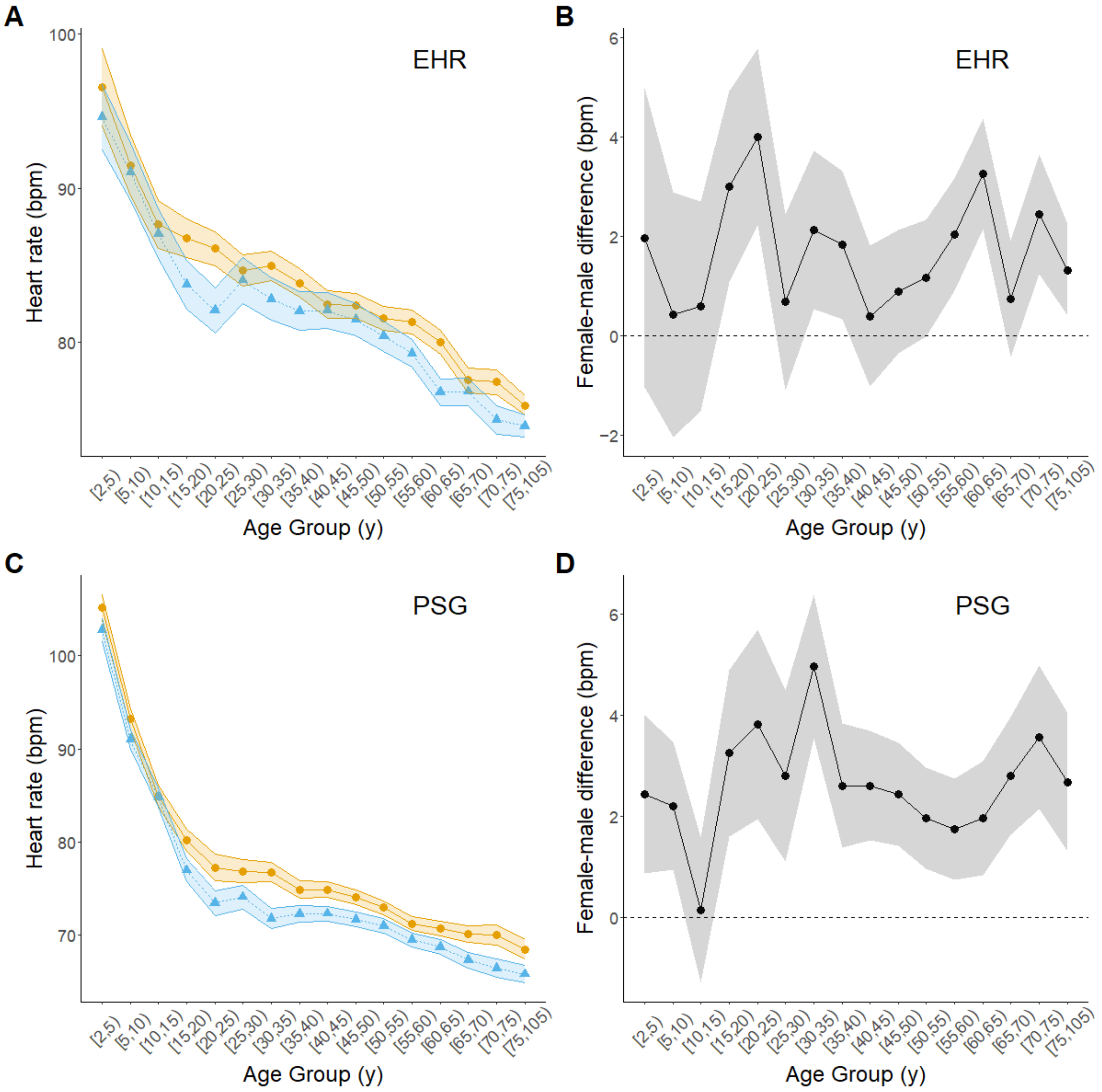

heart rate for men and women from the PSG data set; (**D**). Age-group-specific differences in mean heart rate between women and men from the PSG data set.

Supplementary Figure S4. Estimated age trajectories of respiratory rate for men and women across the life course after adjustment for BMI and disease status. Plotted points represent estimates from fitted regression models for each age group; shaded bands outline 95% upper and lower confidence limits for the estimate at each age group. Amber circles, women; blue triangles, men; black circles, female-minus-male difference. Panels: (**A**). Estimated trajectories of mean respiratory rate for men and women from the EHR data set; (**B**). Age-group-specific differences in mean respiratory rate between women and men from the EHR data set; (**C**). Estimated trajectories of mean respiratory rate for men and women from the PSG data set; (**D**). Age-group-specific differences in mean respiratory rate between women and men from the PSG data set.

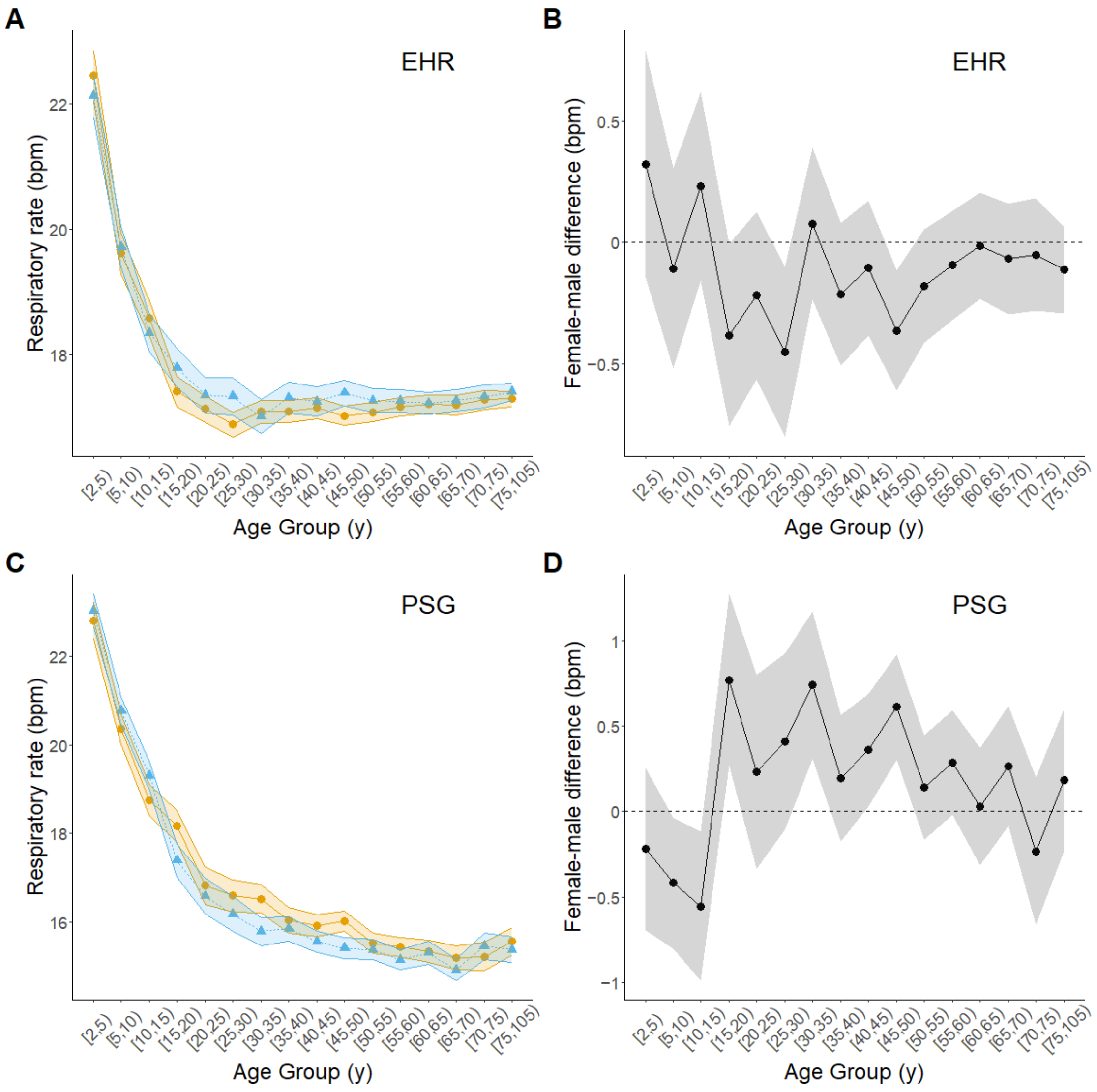

Supplementary Table S1. Counts for the presence (1) and absence (0) of five relevant diseases among the 53,252 subjects in the EHR data where 5,820 subjects (10.93%) had at least one of these diseases, and among the 21,477 subjects in the PSG data where 2,231 subjects (10.39%) had at least one of these diseases. Each column of percents (%) is the percentage of the total number of subjects for the data set.

Coronary Artery Disease	Pulmonary Embolism	Emphysema	Heart Failure	COPD	EHR dataset		PSG dataset	
					count	%	count	%
0	0	0	0	0	47432	89.071	19246	89.612
0	0	0	0	1	973	1.827	642	2.989
0	0	0	1	0	235	0.441	34	0.158
0	0	0	1	1	53	0.100	23	0.107
0	0	1	0	0	1295	2.432	279	1.299
0	0	1	0	1	170	0.319	46	0.214
0	0	1	1	0	37	0.069	2	0.009
0	0	1	1	1	12	0.023	0	0.0
0	1	0	0	0	108	0.203	141	0.657
0	1	0	0	1	6	0.011	11	0.051
0	1	0	1	0	4	0.008	1	0.005
0	1	0	1	1	0	0.0	0	0.0
0	1	1	0	0	12	0.023	11	0.051
0	1	1	0	1	3	0.006	1	0.005
0	1	1	1	0	0	0.0	0	0.0
0	1	1	1	1	0	0.0	1	0.005
1	0	0	0	0	1854	3.482	772	3.595
1	0	0	0	1	234	0.439	102	0.475
1	0	0	1	0	51	0.096	5	0.023
1	0	0	1	1	5	0.009	4	0.019
1	0	1	0	0	530	0.995	110	0.512
1	0	1	0	1	181	0.340	20	0.093
1	0	1	1	0	34	0.064	2	0.009
1	0	1	1	1	7	0.013	3	0.014
1	1	0	0	0	9	0.017	9	0.042
1	1	0	0	1	4	0.008	3	0.014
1	1	0	1	0	0	0.0	0	0.0
1	1	0	1	1	0	0.0	0	0.0
1	1	1	0	0	3	0.006	6	0.028
1	1	1	0	1	0	0.0	3	0.014
1	1	1	1	0	0	0.0	0	0.0
1	1	1	1	1	0	0.0	0	0.0

Supplementary Table S2. Age-group-specific estimates and 95% confidence limits for trajectories of O_2 saturation (%) in the EHR data set (N=23,336) for females and males. Estimates are based on a regression model without adjustment for BMI and correspond to the data displayed in Figure 1a,b.

Age Group	**Female** (N=14,132)					**Male** (N=9,204)					**Female – Male Difference**			
	N	%	Estimate	95% Confidence Limits Lower	95% Confidence Limits Upper	N	%	Estimate	95% Confidence Limits Lower	95% Confidence Limits Upper	Estimate	95% Confidence Limits Lower	95% Confidence Limits Upper	P value
[2,5)	240	1.70	97.7	97.5	98.0	339	3.68	97.7	97.5	97.9	0.00	-0.29	0.29	0.986
[5,10)	296	2.09	98.0	97.8	98.2	323	3.51	97.9	97.7	98.1	0.12	-0.15	0.40	0.773
[10,15)	356	2.52	98.3	98.1	98.4	324	3.52	97.8	97.6	98.0	0.43	0.17	0.69	0.013
[15,20)	487	3.45	98.2	98.1	98.4	288	3.13	98.0	97.8	98.2	0.23	-0.02	0.49	0.320
[20,25)	632	4.47	98.1	98.0	98.3	313	3.40	97.8	97.6	98.0	0.33	0.09	0.57	0.051
[25,30)	722	5.11	98.1	98.0	98.2	295	3.21	97.6	97.4	97.8	0.50	0.26	0.74	0.00041
[30,35)	813	5.75	98.0	97.9	98.0	379	4.12	97.4	97.2	97.6	0.67	0.45	0.88	<0.0001
[35,40)	869	6.15	98.0	97.9	98.0	444	4.82	97.4	97.2	97.6	0.60	0.40	0.80	<0.0001
[40,45)	959	6.79	98.0	97.9	98.1	492	5.35	97.2	97.0	97.4	0.80	0.61	0.99	<0.0001
[45,50)	1195	8.46	97.6	97.5	97.7	639	6.94	97.1	96.9	97.2	0.56	0.39	0.73	<0.0001
[50,55)	1215	8.60	97.4	97.3	97.5	736	8.00	97.0	96.9	97.2	0.40	0.24	0.56	<0.0001
[55,60)	1236	8.75	97.1	97.0	97.2	825	8.96	96.9	96.8	97.0	0.20	0.05	0.36	0.066
[60,65)	1245	8.81	96.9	96.8	97.0	879	9.55	96.8	96.7	96.9	0.14	-0.01	0.29	0.320
[65,70)	1027	7.27	96.7	96.6	96.9	853	9.27	96.6	96.5	96.8	0.11	-0.05	0.27	0.566
[70,75)	1048	7.42	96.7	96.6	96.8	758	8.24	96.3	96.2	96.5	0.33	0.17	0.49	0.00076
[75,105)	1792	12.68	96.3	96.2	96.4	1317	14.31	96.2	96.1	96.3	0.13	0.00	0.25	0.284

Supplementary Table S3. Age-group-specific estimates and 95% confidence limits for trajectories of O_2 saturation (%) in the PSG data set (N=21,477) for females and males. Estimates are based on a regression model without adjustment for BMI and correspond to the data displayed in Figure 1c,d.

Age Group	**Female** (N=10,568)					**Male** (N=10,909)					**Female – Male Difference**			
	N	%	Estimate	95% Confidence Limits		N	%	Estimate	95% Confidence Limits		Estimate	95% Confidence Limits		P value
				Lower	Upper				Lower	Upper		Lower	Upper	
[2,5)	376	3.6	97.5	97.3	97.7	510	4.7	97.5	97.4	97.7	-0.05	-0.29	0.19	1.00
[5,10)	583	5.5	97.9	97.7	98.0	771	7.1	97.8	97.7	97.9	0.09	-0.10	0.29	1.00
[10,15)	456	4.3	98.0	97.9	98.2	586	5.4	97.6	97.5	97.8	0.39	0.17	0.62	0.0048
[15,20)	393	3.7	97.7	97.5	97.9	386	3.5	97.4	97.2	97.5	0.36	0.10	0.62	0.043
[20,25)	285	2.7	97.7	97.5	97.9	323	3.0	97.0	96.8	97.2	0.65	0.36	0.94	0.00012
[25,30)	411	3.9	97.6	97.4	97.8	335	3.1	96.6	96.5	96.8	0.96	0.69	1.22	<0.0001
[30,35)	556	5.3	97.2	97.1	97.4	507	4.6	96.5	96.3	96.6	0.72	0.50	0.94	<0.0001
[35,40)	694	6.6	97.2	97.1	97.3	730	6.7	96.2	96.0	96.3	1.04	0.85	1.23	<0.0001
[40,45)	886	8.4	97.1	96.9	97.2	923	8.5	96.2	96.1	96.3	0.85	0.68	1.02	<0.0001
[45,50)	1,070	10.1	96.6	96.5	96.7	986	9.0	95.9	95.8	96.1	0.62	0.46	0.78	<0.0001
[50,55)	1,151	10.9	96.4	96.3	96.5	982	9.0	95.9	95.8	96.0	0.52	0.37	0.68	<0.0001
[55,60)	1,090	10.3	96.3	96.2	96.4	1,032	9.5	95.8	95.7	95.9	0.43	0.27	0.58	<0.0001
[60,65)	859	8.1	95.9	95.7	96.0	808	7.4	95.6	95.5	95.8	0.23	0.05	0.40	0.064
[65,70)	758	7.2	95.8	95.7	96.0	821	7.5	95.7	95.6	95.9	0.08	-0.10	0.26	1.00
[70,75)	485	4.6	95.7	95.6	95.9	583	5.3	95.9	95.7	96.0	-0.12	-0.34	0.10	1.00
[75,105)	515	4.9	95.6	95.5	95.8	626	5.7	95.8	95.6	95.9	-0.15	-0.36	0.07	0.90

Supplementary Table S4. Age-group-specific estimates and 95% confidence limits for trajectories of serum bicarbonate level (mEq/L) in the EHR data set (N=44,202) for females and males. Estimates are based on a regression model without adjustment for BMI and correspond to the data displayed in Figure 2a,b.

Age Group	**Female** (N=27,294)					**Male** (N=16,628)					**Female – Male Difference**			
	N	%	Estimate	95% Confidence Limits		N	%	Estimate	95% Confidence Limits		Estimate	95% Confidence Limits		P value
				Lower	Upper				Lower	Upper		Lower	Upper	
[2,5)	55	0.20	23.1	22.2	23.9	93	0.56	23.3	22.6	23.9	-0.19	-1.24	0.85	1.00
[5,10)	100	0.37	24.4	23.8	25.1	92	0.55	24.7	24.0	25.3	-0.21	-1.10	0.67	1.00
[10,15)	171	0.63	25.5	25.1	26.0	154	0.92	25.5	25.0	26.0	0.00	-0.68	0.68	1.00
[15,20)	516	1.89	25.2	25.0	25.5	266	1.59	26.9	26.5	27.3	-1.69	-2.15	-1.23	<0.0001
[20,25)	923	3.38	24.8	24.6	25.0	326	1.95	27.1	26.7	27.4	-2.23	-2.62	-1.83	<0.0001
[25,30)	1215	4.45	25.1	24.9	25.2	475	2.84	27.0	26.7	27.2	-1.91	-2.24	-1.58	<0.0001
[30,35)	1528	5.60	25.3	25.2	25.5	609	3.64	27.0	26.8	27.3	-1.67	-1.97	-1.38	<0.0001
[35,40)	1674	6.13	25.6	25.5	25.8	792	4.73	26.9	26.7	27.1	-1.30	-1.56	-1.03	<0.0001
[40,45)	1944	7.12	25.9	25.7	26.0	945	5.65	26.7	26.5	26.9	-0.86	-1.11	-0.62	<0.0001
[45,50)	2387	8.75	26.2	26.1	26.4	1232	7.36	26.6	26.4	26.8	-0.37	-0.58	-0.15	0.0056
[50,55)	2487	9.11	26.8	26.6	26.9	1536	9.18	26.6	26.5	26.8	0.13	-0.07	0.33	0.843
[55,60)	2710	9.93	27.1	27.0	27.2	1802	10.77	26.7	26.5	26.8	0.41	0.22	0.60	0.00013
[60,65)	2714	9.94	27.1	27.0	27.2	1872	11.19	26.9	26.7	27.0	0.26	0.08	0.44	0.031
[65,70)	2591	9.49	27.4	27.3	27.5	1918	11.47	26.9	26.8	27.1	0.49	0.31	0.68	<0.0001
[70,75)	2365	8.66	27.4	27.3	27.6	1651	9.87	27.2	27.0	27.3	0.28	0.08	0.48	0.032
[75,105)	3914	14.34	27.6	27.5	27.7	2965	17.72	27.2	27.1	27.3	0.43	0.28	0.58	<0.0001

Supplementary Table S5. Age-group-specific estimates and 95% confidence limits for trajectories of end-tidal CO_2 level (mmHg) in the PSG data set (N=21,477) for females and males. Estimates are based on a regression model without adjustment for BMI and correspond to the data displayed in Figure 2c,d.

Age Group	**Female** (N=10,568)					**Male** (N=10,909)					**Female – Male Difference**			
	N	%	Estimate	95% Confidence Limits		N	%	Estimate	95% Confidence Limits		Estimate	95% Confidence Limits		P value
				Lower	Upper				Lower	Upper		Lower	Upper	
[2,5)	376	3.6	38.2	37.7	38.7	510	4.7	38.3	37.8	38.7	-0.02	-0.66	0.61	1.00
[5,10)	583	5.5	39.8	39.4	40.2	771	7.1	39.3	38.9	39.6	0.55	0.04	1.07	0.139
[10,15)	456	4.3	39.9	39.5	40.3	586	5.4	39.9	39.6	40.3	-0.05	-0.64	0.53	1.00
[15,20)	393	3.7	39.4	38.9	39.8	386	3.5	40.8	40.3	41.3	-1.44	-2.11	-0.76	0.00022
[20,25)	285	2.7	38.7	38.1	39.2	323	3.0	40.2	39.7	40.7	-1.49	-2.25	-0.73	0.00086
[25,30)	411	3.9	38.8	38.4	39.3	335	3.1	40.7	40.2	41.3	-1.90	-2.59	-1.21	<0.0001
[30,35)	556	5.3	39.2	38.8	39.6	507	4.6	40.8	40.4	41.2	-1.61	-2.19	-1.04	<0.0001
[35,40)	694	6.6	39.5	39.1	39.8	730	6.7	40.5	40.2	40.9	-1.09	-1.58	-0.59	0.00016
[40,45)	886	8.4	39.9	39.6	40.2	923	8.5	40.7	40.4	41.0	-0.83	-1.27	-0.39	0.0013
[45,50)	1,070	10.1	40.2	39.9	40.5	986	9.0	40.1	39.8	40.4	0.12	-0.29	0.54	1.00
[50,55)	1,151	10.9	40.4	40.1	40.7	982	9.0	39.8	39.5	40.1	0.65	0.24	1.06	0.0086
[55,60)	1,090	10.3	40.5	40.2	40.8	1,032	9.5	39.4	39.1	39.6	1.15	0.75	1.56	<0.0001
[60,65)	859	8.1	40.4	40.1	40.7	808	7.4	39.1	38.8	39.4	1.31	0.85	1.77	<0.0001
[65,70)	758	7.2	39.9	39.6	40.2	821	7.5	38.6	38.3	38.9	1.31	0.84	1.78	<0.0001
[70,75)	485	4.6	39.7	39.3	40.1	583	5.3	38.1	37.8	38.5	1.57	1.00	2.15	<0.0001
[75,105)	515	4.9	39.1	38.7	39.5	626	5.7	37.2	36.8	37.6	1.89	1.33	2.45	<0.0001

Supplementary Table S6. Age-group-specific estimates and 95% confidence limits for trajectories of heart rate (bpm) in the EHR data set (N=22,595) for females and males. Estimates are based on a regression model without adjustment for BMI and correspond to the graphs in Figure 3a,b.

	**Female (N=13,724)**					**Male (N=8,871)**					**Female – Male Difference**			
**Age Group**	**N**	**%**	**Estimate**	**95% Confidence Limits**		**N**	**%**	**Estimate**	**95% Confidence Limits**		**Estimate**	**95% Confidence Limits**		P value
				**Lower**	**Upper**				**Lower**	**Upper**		**Lower**	**Upper**	
[2,5)	117	0.85	99.3	97.0	101.6	176	1.98	96.7	94.8	98.6	2.56	-0.46	5.58	0.583
[5,10)	192	1.40	93.0	91.2	94.8	232	2.62	93.2	91.6	94.9	-0.19	-2.66	2.28	1.00
[10,15)	287	2.09	88.4	86.9	89.9	283	3.19	88.3	86.8	89.8	0.11	-2.02	2.23	1.00
[15,20)	452	3.29	87.4	86.2	88.6	275	3.10	84.1	82.6	85.7	3.26	1.33	5.20	0.011
[20,25)	605	4.41	87.3	86.2	88.3	302	3.40	82.8	81.3	84.2	4.49	2.71	6.28	<0.0001
[25,30)	688	5.01	85.5	84.5	86.4	289	3.26	83.2	81.7	84.7	2.30	0.52	4.07	0.079
[30,35)	792	5.77	86.0	85.1	86.9	366	4.13	83.5	82.2	84.8	2.57	0.97	4.17	0.015
[35,40)	850	6.19	85.1	84.2	86.0	433	4.88	82.7	81.4	83.9	2.44	0.94	3.93	0.015
[40,45)	948	6.91	83.6	82.8	84.5	480	5.41	83.1	82.0	84.3	0.49	-0.93	1.91	1.00
[45,50)	1193	8.69	83.1	82.4	83.9	632	7.12	82.3	81.2	83.3	0.90	-0.35	2.14	0.631
[50,55)	1212	8.83	82.8	82.1	83.5	739	8.33	80.9	79.9	81.8	1.93	0.74	3.11	0.015
[55,60)	1240	9.04	82.1	81.4	82.8	828	9.33	79.6	78.7	80.5	2.49	1.35	3.63	0.00023
[60,65)	1241	9.04	81.2	80.5	82.0	891	10.04	77.0	76.2	77.9	4.22	3.11	5.34	<0.0001
[65,70)	1035	7.54	78.4	77.6	79.2	862	9.72	77.5	76.6	78.3	0.94	-0.22	2.11	0.583
[70,75)	1058	7.71	78.1	77.3	78.9	760	8.57	75.2	74.3	76.2	2.87	1.67	4.08	<0.0001
[75,105)	1814	13.22	76.3	75.7	76.9	1323	14.91	74.5	73.8	75.2	1.79	0.87	2.70	0.0015

Supplementary Table S7. Age-group-specific estimates and 95% confidence limits for trajectories of heart rate (bpm) in the PSG data set (N=21,477) for females and males. Estimates are based on a regression model without adjustment for BMI and correspond to the data displayed in Figure 3c,d.

Age Group	**Female** (N=10,568)					**Male** (N=10,909)					**Female – Male Difference**			
	N	%	Estimate	95% Confidence Limits		N	%	Estimate	95% Confidence Limits		Estimate	95% Confidence Limits		P value
				Lower	Upper				Lower	Upper		Lower	Upper	
[2,5)	376	3.6	102.5	101.3	103.7	510	4.7	100.1	99.0	101.1	2.40	0.80	3.99	0.0064
[5,10)	583	5.5	90.8	89.8	91.7	771	7.1	88.5	87.7	89.4	2.25	0.97	3.54	0.0018
[10,15)	456	4.3	83.3	82.2	84.4	586	5.4	83.1	82.2	84.1	0.15	-1.32	1.61	0.844
[15,20)	393	3.7	79.3	78.1	80.4	386	3.5	76 .0	74.8	77.2	3.26	1.58	4.94	0.00058
[20,25)	285	2.7	76.4	75.0	77.8	323	3.0	72.3	70.9	73.6	4.17	2.26	6.08	0.00011
[25,30)	411	3.9	77.4	76.2	78.6	335	3.1	73.4	72.2	74.7	3.95	2.22	5.68	<0.0001
[30,35)	556	5.3	77.4	76.4	78.4	507	4.6	71.6	70.5	72.6	5.80	4.37	7.24	<0.0001
[35,40)	694	6.6	75.6	74.7	76.5	730	6.7	72.1	71.2	73.0	3.47	2.22	4.71	<0.0001
[40,45)	886	8.4	75.6	74.8	76.4	923	8.5	72.4	71.6	73.1	3.26	2.15	4.36	<0.0001
[45,50)	1,070	10.1	75.0	74.2	75.7	986	9.0	71.6	70.8	72.3	3.39	2.35	4.42	<0.0001
[50,55)	1,151	10.9	73.5	72.8	74.2	982	9.0	70.9	70.1	71.6	2.68	1.66	3.70	<0.0001
[55,60)	1,090	10.3	71.7	71.0	72.4	1,032	9.5	69.2	68.4	69.9	2.55	1.53	3.56	<0.0001
[60,65)	859	8.1	70.9	70.1	71.7	808	7.4	68.4	67.6	69.3	2.51	1.36	3.66	0.00011
[65,70)	758	7.2	70.0	69.2	70.9	821	7.5	66.6	65.7	67.4	3.46	2.28	4.65	<0.0001
[70,75)	485	4.6	69.6	68.6	70.7	583	5.3	65.4	64.4	66.3	4.28	2.84	5.72	<0.0001
[75,105)	515	4.9	67.6	66.5	68.6	626	5.7	64.5	63.5	65.4	3.09	1.69	4.49	0.00010

Supplementary Table S8. Age-group-specific estimates and 95% confidence limits for trajectories of respiratory rate (bpm) in the EHR data set (N=18,496) for females and males. Estimates are based on a regression model without adjustment for BMI and correspond to the graphs in Figure 4a,b.

Age Group	Female (N=11,217)					Male (N=7,279)					Female – Male Difference			
	N	%	Estimate	95% Confidence Limits		N	%	Estimate	95% Confidence Limits		Estimate	95% Confidence Limits		P value
				Lower	Upper				Lower	Upper		Lower	Upper	
[2,5)	151	1.35	22.2	21.9	22.6	223	3.06	22.0	21.7	22.3	0.28	-0.19	0.74	1.00
[5,10)	222	1.98	19.5	19.2	19.8	240	3.30	19.5	19.2	19.8	-0.04	-0.46	0.37	1.00
[10,15)	270	2.41	18.4	18.1	18.6	252	3.46	18.1	17.8	18.4	0.28	-0.11	0.67	1.00
[15,20)	380	3.39	17.1	16.9	17.4	221	3.04	17.5	17.2	17.8	-0.35	-0.73	0.02	0.93
[20,25)	489	4.36	16.8	16.6	17.0	250	3.43	17.1	16.8	17.3	-0.25	-0.60	0.10	1.00
[25,30)	566	5.05	16.7	16.5	16.9	223	3.06	17.0	16.7	17.3	-0.31	-0.67	0.04	0.96
[30,35)	632	5.63	17.0	16.8	17.2	295	4.05	16.8	16.6	17.1	0.17	-0.15	0.48	1.00
[35,40)	689	6.14	16.9	16.7	17.1	338	4.64	17.1	16.9	17.3	-0.18	-0.48	0.11	1.00
[40,45)	753	6.71	17.0	16.9	17.2	388	5.33	17.2	16.9	17.4	-0.13	-0.40	0.15	1.00
[45,50)	960	8.56	16.9	16.7	17.0	494	6.79	17.1	16.9	17.3	-0.26	-0.51	-0.02	0.56
[50,55)	958	8.54	17.0	16.9	17.1	587	8.06	17.2	17.0	17.4	-0.21	-0.45	0.02	0.94
[55,60)	1002	8.93	17.0	16.9	17.2	651	8.94	17.2	17.0	17.4	-0.16	-0.38	0.07	1.00
[60,65)	991	8.83	17.2	17.0	17.3	701	9.63	17.2	17.0	17.3	0.02	-0.20	0.24	1.00
[65,70)	838	7.47	17.1	16.9	17.2	700	9.62	17.2	17.1	17.4	-0.13	-0.36	0.09	1.00
[70,75)	869	7.75	17.2	17.1	17.4	638	8.76	17.1	16.9	17.3	0.15	-0.08	0.38	1.00
[75,105)	1447	12.90	17.1	17.0	17.3	1078	14.81	17.4	17.2	17.5	-0.22	-0.40	-0.04	0.24

Supplementary Table S9. Age-group-specific estimates and 95% confidence limits for trajectories of respiratory rate (bpm) in the PSG data set (N=21,477) for females and males. Estimates are based on a regression model without adjustment for BMI and correspond to the data displayed in Figure 4c,d.

Age Group	**Female** (N=10,568)					**Male** (N=10,909)					**Female – Male Difference**			
	**N**	**%**	**Estimate**	**95% Confidence Limits**		**N**	**%**	**Estimate**	**95% Confidence Limits**		**Estimate**	**95% Confidence Limits**		**P value**
				**Lower**	**Upper**				**Lower**	**Upper**		**Lower**	**Upper**	
[2,5)	376	3.6	21.9	21.5	22.2	510	4.7	22.1	21.8	22.4	-0.23	-0.71	0.25	1.00
[5,10)	583	5.5	19.5	19.2	19.8	771	7.1	19.9	19.7	20.2	-0.40	-0.79	-0.01	0.28
[10,15)	456	4.3	18.2	17.9	18.5	586	5.4	18.7	18.5	19.0	-0.55	-0.99	-0.10	0.14
[15,20)	393	3.7	17.9	17.5	18.2	386	3.5	17.1	16.7	17.4	0.77	0.26	1.28	0.04
[20,25)	285	2.7	16.6	16.1	17.0	323	3.0	16.2	15.8	16.6	0.33	-0.25	0.91	1.00
[25,30)	411	3.9	16.7	16.4	17.1	335	3.1	16.0	15.6	16.4	0.75	0.22	1.27	0.06
[30,35)	556	5.3	16.7	16.4	17.0	507	4.6	15.7	15.4	16.0	0.98	0.54	1.41	0.0002
[35,40)	694	6.6	16.2	16.0	16.5	730	6.7	15.8	15.5	16.1	0.44	0.06	0.82	0.18
[40,45)	886	8.4	16.1	15.9	16.4	923	8.5	15.6	15.3	15.8	0.55	0.21	0.88	0.02
[45,50)	1,070	10.1	16.3	16.1	16.5	986	9.0	15.4	15.2	15.6	0.89	0.58	1.20	<0.0001
[50,55)	1,151	10.9	15.7	15.5	15.9	982	9.0	15.3	15.1	15.6	0.34	0.03	0.65	0.21
[55,60)	1,090	10.3	15.6	15.4	15.8	1,032	9.5	15.1	14.8	15.3	0.52	0.21	0.83	0.01
[60,65)	859	8.1	15.4	15.2	15.6	808	7.4	15.2	15.0	15.5	0.19	-0.16	0.54	1.00
[65,70)	758	7.2	15.2	14.9	15.4	821	7.5	14.7	14.5	15.0	0.45	0.10	0.81	0.13
[70,75)	485	4.6	15.1	14.8	15.4	583	5.3	15.1	14.8	15.4	-0.03	-0.47	0.40	1.00
[75,105)	515	4.9	15.3	15.0	15.6	626	5.7	15.0	14.7	15.3	0.30	-0.12	0.72	0.83
